# In Vivo Assessment of Anti-Inflammatory Effects of Aqueous Extracts of *Nepeta nuda* ssp. *nuda* L. in Experimental Model of Peripheral Inflammation in Male Long Evans Rats

**DOI:** 10.3390/life15121938

**Published:** 2025-12-18

**Authors:** Milena Keremidarska-Markova, Veneta Evtimova-Koeva, Tsvetozar Penchev, Dilyana Doncheva-Stoimenova, Miroslava Zhiponova, Mariela Chichova, Bilyana Ilieva

**Affiliations:** 1 Faculty of Biology, Sofia University St. Kliment Ohridski, 1164 Sofia, Bulgariadonchevast@biofac.uni-sofia.bg (D.D.-S.); zhiponova@biofac.uni-sofia.bg (M.Z.); b.ilieva@biofac.uni-sofia.bg (B.I.); 2 Centre of Competence “Sustainable Utilization of Bio-resources and Waste of Medicinal and Aromatic Plants for Innovative Bioactive Products” (BIORESOURCES BG), 1000 Sofia, Bulgaria

**Keywords:** *Nepeta nuda*, carrageenan edema, inflammation, inflammatory pain, locomotor activity

## Abstract

**Background**: Recently various plants have attracted considerable scientific interest as potential therapeutic alternatives to known drugs used in anti-inflammatory therapy. Therefore, we have investigated the possible anti-inflammatory and analgesic effects of aqueous extracts from flowers of the medical plant *Nepeta nuda* ssp. *nuda* L. (naked catmint) in a model of acute peripheral inflammation induced by intraplantar injection of λ-carrageenan in the hind paw of Long Evans rats. **Methods**: Two routes of *N. nuda* extract application were used: locally by intraplantar injections at dosages of 2.5 mg/kg and 25 mg/kg and systemically by intraperitoneal administration at dosages of 50 mg/kg and 200 mg/kg, respectively. Paw volume was measured prior to the carrageenan application and 1, 2, 3, and 4 h after carrageenan injection. Spontaneous locomotor activity of the rats was assessed 3 h after carrageenan injection, corresponding to the peak of acute paw inflammation. **Results**: Local application of the higher *N. nuda* dose led to a marked reduction in inflammatory paw edema at the 4th hour after carrageenan injection, comparable to the effect of the positive control diclofenac sodium. Interestingly, a similar anti-inflammatory effect was observed at the 1st hour when both extract doses were administered intraperitoneally. Only the higher intraplantar dose of *N. nuda* extract significantly enhanced the vertical activity in comparison to the group treated with carrageenan alone. **Conclusions**: Our data indicate that the aqueous *N. nuda* extract possesses potent anti-inflammatory effects following both local and systemic administration in rats. Furthermore, when administered locally the extract exerts significant analgesic activity in inflammatory pain.

## 1. Introduction

Inflammation is a natural biological response to various forms of injury or infection and acts as an innate defense mechanism of the body [1,2]. This multi-step process not only aims to eliminate the cause that triggered the inflammatory process but also initiates tissue repair and regeneration [3]. Despite its initial protective role, inflammation can cause serious health disorders leading to morbidity and even mortality [4,5,6].

Inflammation can be divided into two main types: acute and chronic. Acute inflammation is short-lived and characterized by swelling, redness and pain, whereas chronic inflammation persists for long periods and often results in progressive tissue destruction and the development of diseases such as arthritis, diabetes and cardiovascular disorders [7]. Although peripheral nonspecific inflammation is acute in nature, it may overlap with chronic inflammation in terms of mechanisms and duration [8]. Understanding the mechanisms of peripheral nonspecific and chronic inflammation reveals important similarities fundamental to their pathophysiology. Both types of inflammation involve an impaired immune response. Oxidative stress also plays an important role in the inflammatory response, often reducing the phagocytic activity of leukocytes through both oxygen-dependent and oxygen-independent mechanisms [9].

Edema is an early sign of inflammation and is caused by transcapillary flow of protein-rich fluid into the interstitium as a result of the actions of histamine, bradykinin, leukotrienes, components of the complement, substance P, and platelet-activating factor [10,11]. These mediators significantly alter the barrier functions of small blood vessels and increase the permeability of capillaries and venules to both water and proteins [1].

Modern medicine offers a variety of drugs for the treatment of peripheral nonspecific and chronic inflammation, including: nonsteroidal anti-inflammatory drugs (NSAIDs), which inhibit the cyclooxygenase (COX) enzyme and suppress the production of prostaglandins; corticosteroids, which suppress the immune system and inflammatory mediators; and biological agents such as monoclonal antibodies targeting specific cytokines (TNF-α, etc.). Although effective, these drugs often carry side effects and long-term risks, necessitating the search for alternative treatment approaches [12]. Consequently, many plant species are currently being investigated as potential sources for therapeutic alternatives to conventional anti-inflammatory drugs, as they commonly exhibit fewer side effects and better tolerance [13].

For centuries, numerous species from the *Lamiaceae* family have been used due to their well-known antioxidant, antimicrobial, antiviral, antiseptic, anticancer and analgesic properties [14,15]. Multiple studies have shown that the genus *Nepeta*, rich in biologically active compounds such as terpenoids and phenolic constituents, exhibits anti-inflammatory, anticarcinogen, antioxidant, anticonvulsant, antinociceptive and antidepressant activity [16,17,18]. In vitro studies have demonstrated that aqueous extracts from *N. nuda* L. exert antiviral, anticancer and anti-inflammatory effects, along with antioxidant protection for the cells [19,20,21,22,23]. The anti-inflammatory activity may be associated with the presence of flavonoids, nepetalactones and terpenoids, which can modulate key inflammatory mediators [24,25]. However, the exact mechanisms involved and the potential effect on the whole organism remain to be elucidated.

Therefore, in the present study we aimed to investigate the in vivo anti-inflammatory effects of aqueous extract from *N. nuda*, administered via two routes—locally and intraperitoneally, using a carrageenan-induced peripheral inflammation model in the rat hind paw. This model is considered one of the most suitable for evaluating the potential anti-inflammatory activity of test compounds [26,27]. Additionally, the rats’ spontaneous locomotor activity was assessed, as changes in spontaneous activity represent an objective indicator of pain-related behavior that can be reversed by analgesic and anti-inflammatory drugs [28,29].

## 2. Materials and Methods

### 2.1. Chemicals

Sodium chloride (NaCl), diclofenac sodium, and λ-carrageenan were purchased by Sigma-Aldrich Inc. (St. Louis, MO, USA). Sterile saline solution (0.9% NaCl) was obtained from B. Braun Melsungen AG (Melsungen, Germany).

Fresh stock solutions of diclofenac sodium and λ-carrageenan were prepared prior to each experiment in 0.9% NaCl saline.

### 2.2. Plant Material and Preparation and Composition of Plant Extracts

*Nepeta nuda* subsp. *nuda* L. plants were collected from their natural habitat in Bekovi Skali (at approximately 1320 m a.s.l.), Rhodope Mountains [41.99437188774722, 24.396310265460215], Bulgaria, during the flowering period. A voucher specimen (SO108229) has been deposited in the Herbarium of Sofia University “St. Kliment Ohridski,” Sofia, Bulgaria.

The *N. nuda* plants were air-dried in the dark at room temperature to a constant weight, after which the flowers were ground separately into a fine powder. Flower material (1 g in 10 mL) was extracted with water by maceration at 40 °C for 24 h. The resulting aqueous extracts were filtered through filter paper, and the solvent was removed by freezing followed by lyophilization using a freeze-dryer (Alpha 1-2 LDplus, Martin Christ Gefriertrocknungsanlagen GmbH, Osterode am Harz, Germany) at −65 °C. The dried extract was then dissolved in 0.9% NaCl to a known concentration. The extract was kept at −20 °C before administration to preserve its stability.

Primary and secondary metabolites of *N. nuda* flowers were previously identified using GC-MS analysis on a system of an Agilent GC 7890 gas chromatograph and an Agilent MD 5975C mass spectral detector (Supplementary Materials, Table S1; [22]). Secondary metabolites were also identified using Orbitrap-MS^n^ analysis in the negative ionization mode of the UHPLC-LTQ OrbiTrap MS instrument (Supplementary Materials, Table S2; [20]). Metabolites in aqueous extracts from *N. nuda* flowering plants were analyzed by NMR (Supplementary Materials, Table S3; [19]). Quantification of extract yield and total phenolics and flavonoids in plant organs was performed according to [20] (Table 1).

Data for the in vitro biological activity (cytotoxicity and antiviral, antioxidant, and anti-inflammatory activities) of aqueous extract from *N. nuda* flowers were also previously obtained (Supplementary Materials, Table S4; [20,22]).

### 2.3. Animals

In the present study we have used male Long Evans rats (360 ± 12 g body weight) while female animals were excluded from the experimental design in order to avoid possible cyclic hormonal changes that could increase experimental variables and compromise the results. were obtained from Vivarium with Physiological Laboratory as part of the Centre of Competence “Sustainable Utilization of Bio-resources and Waste of Medicinal and Aromatic Plants for Innovative Bioactive Products” (BIORESOURCES BG), project BG16RFPR002-1.014-0001, at the Faculty of Biology of Sofia University “St. Kliment Ohridski”. They were housed in polycarbonate cages with steel wire tops, with six animals kept per cage and provided a standard pelleted diet (TopMix^®^ Laboratory Animals, HL-TopMix Ltd., Sliven, Bulgaria) and water ad libitum. Housing conditions were maintained at temperature of 22 ± 2 °C, 50–60% humidity, and 12:12 h light-dark cycle. During the whole experiment, animal behavior and health conditions were monitored.

All experimental procedures were strictly performed in accordance with the regulations outlined in Directive 2010/63/EU of the European Parliament and of the Council of 22 September 2010 regarding the protection of animals used for scientific purposes. Furthermore, the research was conducted under permit No. 381 from 12 March 2024, issued by the Bulgarian Food Safety Agency under the Ministry of Agriculture, Food, and Forestry.

### 2.4. Experimental Protocol and Groups

**Figure 1:**
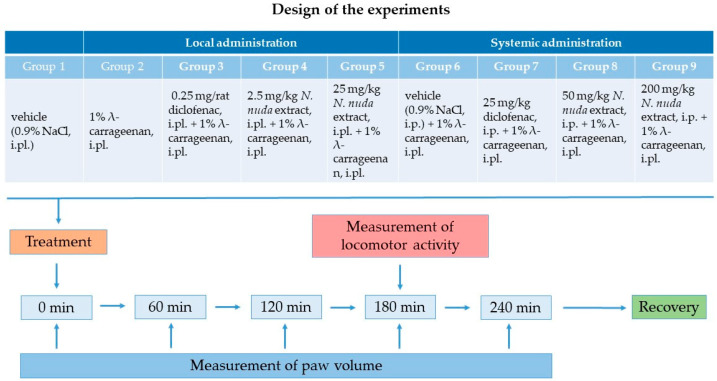
Diagram of the experimental design. i.pl.—intraplantarly; i.p.—intraperitoneally.

The overall experimental design is presented in Figure 1.

Edema was induced in the right hind paw of each rat by intraplantar injection of a carrageenan suspension [30] (1% *w*/*v* λ-carrageenan in normal saline) in a volume of 100 μL except for the control group. Instead, 100 μL of sterile saline was intraplantarly injected into the rat hind paw of the rats from the control group.

The applied concentrations of *N. nuda* aqueous extract were based on previous in vitro studies showing 100% inhibitory anti-inflammatory activity at 2 mg/mL (as shown by the microtiter hemolytic complement assay) [21,22]. The *N. nuda* extract (stock solutions prepared in normal saline) was administered via two routes. Local administration was performed by intraplantar injections of 100 μL of plant extract at doses of 2.5 mg/kg (corresponding to a concentration of 10 mg/mL) or 25 mg/kg (corresponding to a concentration of 100 mg/mL) into the carrageenan-injected hind paw of the rats. In the positive control group, diclofenac instead of plant extract was used as a standard anti-inflammatory drug and administered intraplantarly at a dosage of 0.25 mg/rat. For systemic administration rats received single intraperitoneal injections of 500 μL of the plant extract at doses of 50 mg/kg or 200 mg/kg, applied prior to carrageenan injections. These selected doses corresponded to those used in other similar studies [17,31]. Comparable volumes of 500 μL of either vehicle (sterile saline) or diclofenac (a dosage of 25 mg/kg) were administered intraperitoneally in the placebo and positive control groups, respectively.

The rats (total number of 54) were randomly divided into nine groups of six animals each as follows:

1. Groups with carrageenan-induced paw edema with intraplantar (local) administration of anti-inflammatory agents:
  - Control group with intraplantar injection of vehicle (0.9% sterile normal saline) into the hind paw of rats to eliminate the possible effect of the injection itself; this group was common to both routes of application;
  - A group with λ-carrageenan (1% *w*/*v* carrageenan in 0.9% normal saline) intraplantarly injected into the hind paw of rats;
  - A positive control group with local administration of diclofenac as a standard anti-inflammatory drug (0.25 mg/rat), intraplantarly injected into the carrageenan-treated hind paw of rats;
  - Two groups with local administration of *N. nuda* extract intraplantarly injected into the carrageenan-treated hind paw at doses corresponding to 2.5 mg/kg or 25 mg/kg, respectively.
2. Groups with carrageenan-induced paw edema with systemic administration of anti-inflammatory reagents:
  - A placebo group with intraperitoneal injection of vehicle (0.9% sterile normal saline);
  - A positive control group with intraperitoneal injection of diclofenac at a dosage of 25 mg/kg;
  - Two groups with *N. nuda* extract intraperitoneally injected at doses of 50 mg/kg or 200 mg/kg, respectively.

### 2.5. Measurement of Carrageenan-Induced Paw Edema

The volume of the injected paw was measured prior to the carrageenan application and 1, 2, 3, and 4 h after carrageenan injection, using a digital Plethysmometer apparatus (Model No 37140, Ugo Basile^®^, Ugo Basile S.R.L., Gemonio, Italy).

The initial paw volume before carrageenan injection was considered as a control volume for each animal and percentage changes after treatment were determined and used to plot time course curves. The areas under the time course curves (AUCs) were calculated in arbitrary units to assess the extent of the total paw edema [32]. To examine the anti-inflammatory effect of *N. nuda* extracts the change in edema was calculated as percentages from the mean paw volume of the group treated with carrageenan alone or the placebo group at the corresponding time points set to 100% in local or systemic routes of application, respectively.

### 2.6. Measurement of Spontaneous Motor Activity

Spontaneous locomotor activity of the rats was assessed 3 h after carrageenan application, when the peak of the paw edema was observed. The animals were not habituated to the testing cage prior to the activity assessment in order to measure their locomotor activity during the exploration phase in a novel environment. The rats were individually placed into Multiple Activity Cage apparatus (Model No 47420, Ugo Basile^®^, Ugo Basile S.R.L., Gemonio, Italy) and their horizontal and vertical activity (rearing) were automatically tracked over a 5 min period.

The following two behavioral variables were measured and analyzed: (a) horizontal activity—the total number of interruptions of the horizontal sensors, equivalent to the ambulatory activity at the floor level of the cage and small movement performance (e.g., grooming), and (b) vertical activity—the total number of interruptions of the vertical sensors, assessing rearing and high sniffing activities.

Silent environment was strongly maintained during the experimental period. At the end of the tracking session, each rat was returned to its home cage and the chamber was thoroughly cleaned with ethyl alcohol (70% *v*/*v*) to remove any olfactory cues that might affect the next animal.

### 2.7. Statistical Analysis

The data obtained are presented as mean values ± standard error of the mean (SEM). One-Way Analysis of Variance was employed for intergroup comparisons followed by post hoc analysis with a Tukey test for multiple comparisons. For comparison of results obtained from a single animal during the edema progression, Paired samples *t*-test was used. A value of *p* < 0.05 was considered significant. All statistical analyses were computed using SigmaPlot version 11.0.

## 3. Results

### 3.1. Inhibition of Carrageenan-Induced Paw Edema by Local Administration of N. nuda in Rat

**Figure 2:**
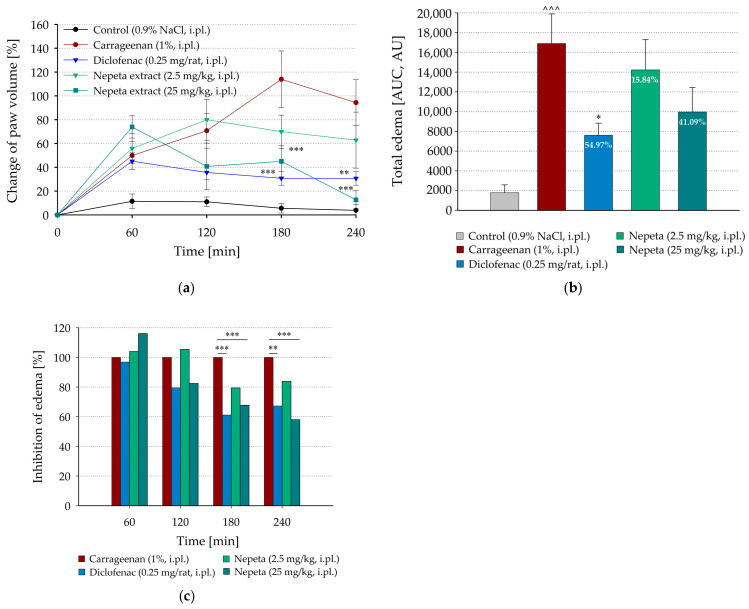
Effect of intraplantarly administrated *N. nuda* extract on carrageenan-induced paw edema. (**a**) Time course changes in paw volume before and 1, 2, 3, and 4 h after carrageenan application; (**b**) Effect of *N. nuda* extract on the total edema calculated in arbitrary units (AU) as areas under the corresponding time course curves (AUC); (**c**) Changes in carrageenan-induced paw edema calculated as percentages from the mean paw volumes of group treated with carrageenan alone at the corresponding time points. Diclofenac (0.25 mg/rat) was used as a positive control. Data are plotted as mean ± SEM of six animals. Numbers in the bars represent the inhibitory effects of diclofenac and *N. nuda* extract calculated as percentages from AUC of carrageenan-treated group. Asterisks indicate significant differences from the group treated with carrageenan alone: * *p* < 0.05, ** *p* < 0.01, *** *p* < 0.001. Carets indicate significant differences from the control group intraplantarly injected with saline: ^˄˄˄^ < 0.001. i.pl.—intraplantarly.

**Figure 3:**
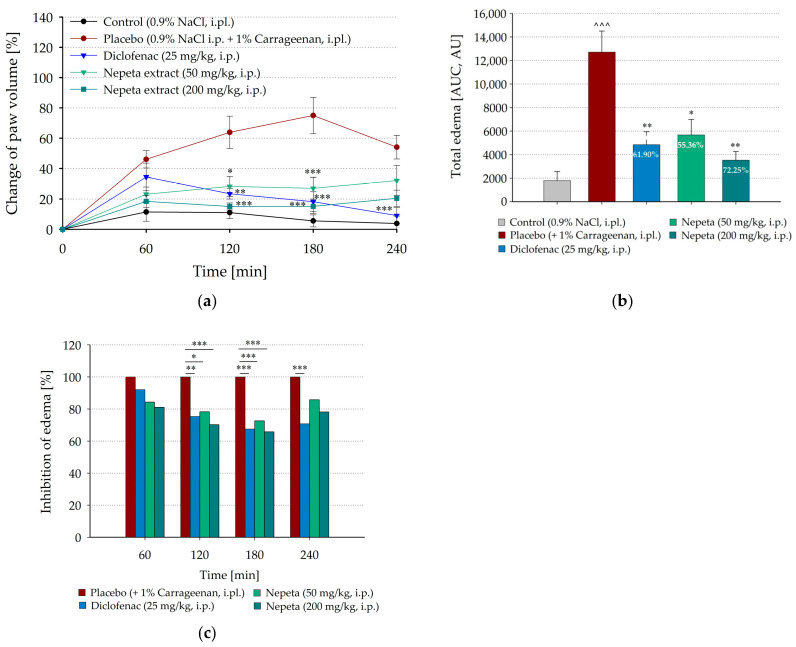
Effect of intraperitoneally administrated *N. nuda* extract on carrageenan-induced paw edema. (**a**) Time course changes in paw volume before and 1, 2, 3, and 4 h after carrageenan application; (**b**) Effect of *N. nuda* extract on the total edema calculated in arbitrary units (AU) as areas under the corresponding time course curves (AUC); (**c**) Changes in carrageenan-induced paw edema calculated as percentages from the mean paw volumes of placebo group treated with carrageenan at the corresponding time points. Diclofenac (25 mg/kg) was used as a positive control. Data are plotted as mean ± SEM of six animals. Numbers in the bars represent the inhibitory effects of diclofenac and *N. nuda* extract calculated as percentages from AUC of placebo group treated with carrageenan. Asterisks indicate significant differences from the placebo group treated with carrageenan: * *p* < 0.05, ** *p* < 0.01, *** *p* < 0.001. Carets indicate significant differences from the control group intraplantarly injected with saline: ^˄˄˄^ < 0.001. i.pl.—intraplantarly; i.p.—intraperitoneally.

A carrageenan-induced paw edema model was used to evaluate the possible anti-inflammatory effect of *N. nuda* extract applied via two routes—locally by intraplantar injection at doses of 2.5 mg/kg or 25 mg/kg into the carrageenan-injected hind paw of the rats, and by intraperitoneal administration at doses of 50 mg/kg or 200 mg/kg to test the systemic action of the plant extract. As shown in Figure 2a and Figure 3a, in the groups treated with carrageenan, acute paw inflammation was markedly induced by carrageenan injection into the hind paw (Table 2) with a progressive increase in the paw volume up to 3 h after administration, followed by a decrease at the 4-th hour. In contrast, no significant change in the paw volume in the control animals, injected intraplantarly with saline, was observed (by 11.41 ± 6.10%, *p* = 0.127, and 11.01 ± 4.00%, *p* = 0.05, at the 1st and 2nd hours after injection, respectively), thereafter the volume recovered probably due to resorption of the saline.

The carrageenan-induced paw edema was significantly inhibited by the intraplantar administration of *N. nuda* extract at a dose of 25 mg/kg at the 4th hour of carrageenan injection (*p* < 0.001, Figure 2a), whereas the lower dose of 2.5 mg/kg showed a little statistically insignificant effect. To assess the extent of the total carrageenan-induced paw edema the areas under the time course curves were obtained and the inhibitory effects of diclofenac and *N. nuda* extract were calculated as percentages from AUC of group treated with carrageenan alone. The total carrageenan-induced edema (Figure 2b) was significantly suppressed only by diclofenac (54.97% inhibitory effect, *p* = 0.020), while the results for *N. nuda* extracts were statistically insignificant (15.84% and 41.09% inhibitory effects at doses of 2.5 and 25 mg/kg, respectively). Data for the time course of inflammation showed that significant effects of diclofenac on paw edema were evident from the 3rd hour after carrageenan injection. However, *N. nuda* extract at a dose of 25 mg/kg was more efficient in reducing paw edema at the 4th hour of carrageenan injection compared to the diclofenac effect (57.98%, *p* < 0.001, vs. 67.26%, *p* = 0.001, from the mean paw volume of the group treated with carrageenan alone, respectively, Figure 2c).

### 3.2. Inhibition of Carrageenan-Induced Paw Edema by Systemic Administration of N. nuda in Rat

The systemic anti-inflammatory effects of *N. nuda* extracts were also examined through intraperitoneal administration (Figure 3). In both doses of 50 mg/kg or 200 mg/kg, the extracts inhibited paw edema, as the effect tended to increase by the 3rd hour after carrageenan injection (up to 72.63%, *p* < 0.001, and 65.79%, *p* < 0.001, from the mean paw volume of the placebo group treated with carrageenan, respectively, Figure 3a,c) and became weaker at the 4th hour (85.77%, *p* = 0.978, and 78.19%, *p* = 0.099, respectively). The results from AUC calculations confirmed the significant inhibition of the total carrageenan-induced edema after intraperitoneal administration of diclofenac, as well as of *N. nuda* extract in both tested doses (Figure 3b). Moreover, the effect of the plant extracts at dose of 200 mg/kg was stronger than that of diclofenac (72.25% inhibitory effect, *p* = 0.002, vs. 61.90% inhibitory effect, *p* = 0.009, respectively). The decrease in paw edema at the 1st hour after carrageenan injection was more pronounced in the animals treated with both doses of *N. nuda* extracts (84.33% and 81.09% from the mean paw volume of the placebo group treated with carrageenan, for 50 and 200 mg/kg, respectively, Figure 3a,c) than in the group, treated with diclofenac (92.10% from the mean paw volume of the placebo group treated with carrageenan), however these inhibitory effects were not statistically significant. However, at the 4th hour diclofenac was more efficient (70.80%, *p* < 0.001, from the mean paw volume of the placebo group treated with carrageenan. Figure 3a,c) than the plant extracts.

### 3.3. Measurement of N. nuda Effects on the Spontaneous Motor Activity During Carrageenan-Induced Inflammation in Rat

**Figure 4:**
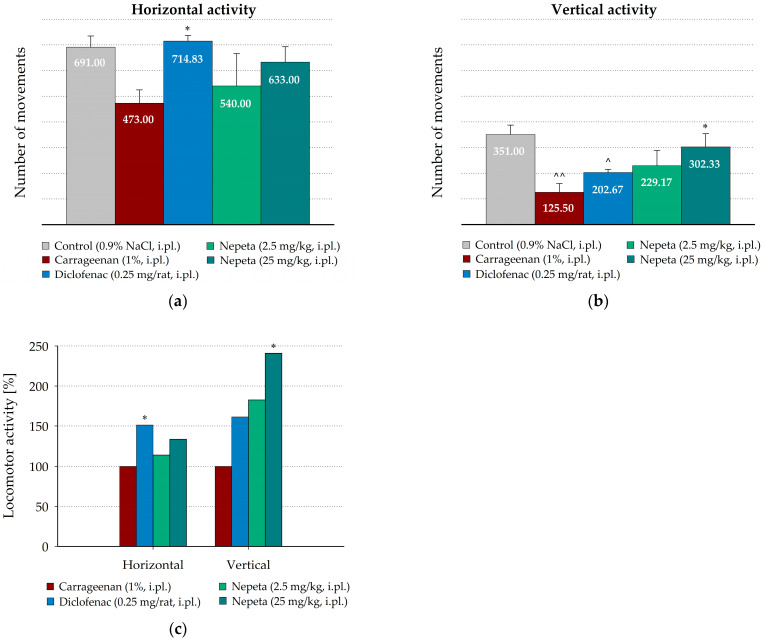
Effect of intraplantarly administrated *N. nuda* extract on locomotor activity in carrageenan-induced paw edema. Horizontal (**a**) and vertical (**b**) activity was measured 3 h after carrageenan application; (**c**) Changes in locomotor activity calculated as percentages from the number of movements of the group treated with carrageenan alone. Diclofenac (0.25 mg/rat) was used as a positive control. Data are plotted as mean ± SEM of six animals. Asterisks indicate significant differences from the group treated with carrageenan alone: * *p* < 0.05. Carets indicate significant differences from the control group intraplantarly injected with saline: ^˄^ < 0.05, ^˄˄^ < 0.01. i.pl.—intraplantarly.

Spontaneous locomotor activity of the rats was assessed 3 h after carrageenan injection, when the peak of acute paw inflammation was observed. The results for the motor activity of the rats with local application of *N. nuda* extracts are shown in Figure 4. Carrageenan-induced edema decreased the number of horizontal movements compared to the control animals, intraplantarly injected with saline (473.00 ± 51.66 vs. 691.00 ± 43.42, Figure 4a). The number of the vertical movements was more strongly influenced (125.50 ± 34.19 vs. 351.00 ± 36.26, *p* = 0.001, Figure 4b). The rats locally treated with *N. nuda* extract at both tested doses showed an increase in the number of horizontal movements compared to the group treated with carrageenan alone (114.16% and 133.83% from the number of movements of the group, treated with carrageenan alone, for doses of 2.5 mg/kg and 25 mg/kg, respectively, Figure 4c). However, these differences did not reach statistical significance.

In contrast, diclofenac, locally applied at dose of 0.25 mg/rat, significantly increased the number of horizontal movements (151.13%, *p* = 0.041, from the number of those of the group treated with carrageenan alone, Figure 4c). However, *N. nuda* extract at dose of 25 mg/kg significantly enhanced the vertical activity in comparison to the group treated with carrageenan alone (240.90%, *p* = 0.012, from the number of group’s treated with carrageenan alone, Figure 4c), while its lower dose, as well as diclofenac, had weaker, statistically insignificant effects.

**Figure 5:**
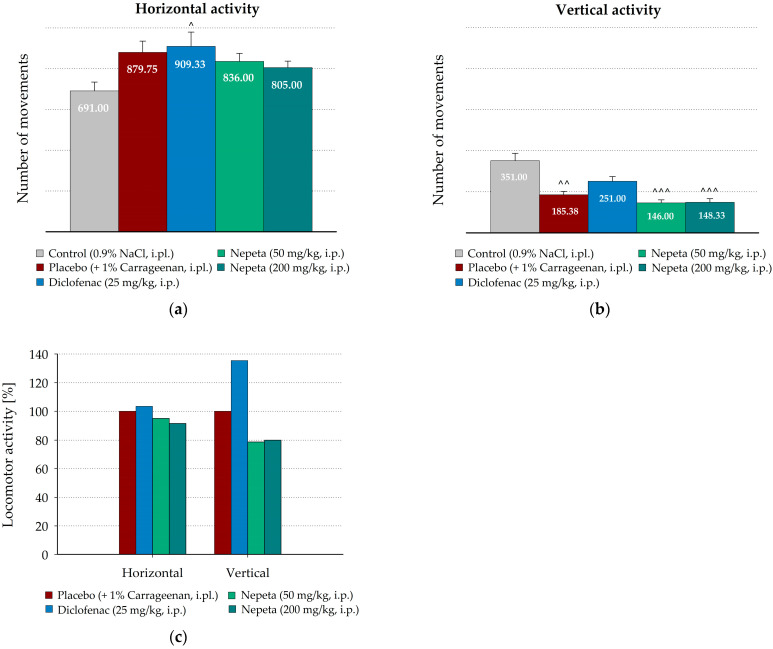
Effect of intraperitoneally administrated *N. nuda* extract on locomotor activity in carrageenan-induced paw edema. Horizontal (**a**) and vertical (**b**) activity was assessed 3 h after carrageenan application; (**c**) Changes in locomotor activity calculated as percentages from the placebo group treated with carrageenan. Diclofenac (25 mg/kg) was used as a positive control. Data are plotted as mean ± SEM of six animals. Carets indicate significant differences from the control group intraplantarly injected with saline: ^˄^ < 0.05, ^˄˄^ < 0.01, ^˄˄˄^ < 0.001. i.pl.—intraplantarly; i.p.—intraperitoneally.

The effects of intraperitoneal administration of *N. nuda* extracts on the spontaneous locomotor activity of the rats were also assessed (Figure 5). Interestingly, in the placebo group, intraperitoneally treated with saline, carrageenan application non-significantly increased the number of horizontal movements compared to the control animals intraplantarly injected with saline (Figure 5a). Similar increased horizontal activity was measured in the groups intraperitoneally treated with both doses of *N. nuda* extract, as well as in diclofenac-treated rats. Non-significant differences in the number of horizontal movements were registered between *N. nuda* extract- and diclofenac-treated groups and the group treated with carrageenan alone (Figure 5c).

However, carrageenan-induced edema significantly decreased the number of vertical movements compared to the control animals intraplantarly injected with saline (185.38 ± 15.90 vs. 351.00 ± 36.26, *p* = 0.002, Figure 5b). As shown in Figure 5c, *N. nuda* extract, intraperitoneally applied at both tested doses, did not influence the vertical activity compared to the placebo group, treated with carrageenan, while the intraperitoneally applied diclofenac non-significantly increased the number of vertical movements (135.40% from the number of group treated with carrageenan alone).

## 4. Discussion

Carrageenan-induced paw edema is one of the most widely used tests for screening biologically active substances with potential anti-inflammatory activity [30]. It is well established as a valid model for the assessment of NSAIDs’ activity [33]. The development of edema following carrageenan injection in the paw is described as a two-step process in which different mediators act sequentially to induce this inflammatory response [34,35]. The initial phase of edema (0–1 h), which is not inhibited by NSAIDs, is associated with the release of histamine and 5-hydroxytryptamine, followed by the production of bradykinin from kallikrein [36,37]. In contrast, the second phase of increasing swelling and redness in the hind paw (1–6 h) is related to increased prostaglandin production by cyclooxygenases (COX-1 and COX-2 enzymes) [35,38].

The species *N. nuda* has been used as a medicinal plant in traditional Bulgarian folk medicine for its beneficial effects on human health due to its antioxidant, antiviral, antibacterial potential, along with anti-inflammatory effects. Comparison of different plant organs indicated that both the flowers and leaves of wild-grown *N. nuda* represent promising sources of anti-inflammatory agents ([20], Table 1). The high biological activities of *N. nuda* flowers have been largely attributed to their enriched content of phenolics, anthocyanins, iridoids, and sugars [20,22]. The in vitro anti-inflammatory activity showed significant correlation with the anthocyanin and flavonoid content, as well as the presence of citramalic acid, myo-inositol, sucrose, hydroquinone, homovanillyl alcohol [22]. Growing evidence suggests that whole plant extracts often exhibit stronger bioactive effects than isolated phytochemicals [39], largely due to interactions among the numerous constituents within the extracts. These interactions may include synergistic or additive effects among secondary metabolites, as well as the ability of complex mixtures to modulate multiple molecular targets simultaneously. Use of water as the extraction solvent resulted in the highest yield of *N. nuda* extract, characterized by a high content of phenolic antioxidants [20].

In the present in vivo study, the dosages of *N. nuda* flower extract—2.5 mg/kg and 25 mg/kg for local application and 50 mg/kg and 200 mg/kg for systemic administration, were selected based on previously conducted in vitro studies of anti-inflammatory activity in cell cultures [22], as well as currently available data. Our results demonstrated a significant inhibition of carrageenan-induced paw edema in rats following either local or systemic administration of the aqueous *N. nuda* extract. In addition, our results suggest that the intraperitoneal administration of *N. nuda* may produce a more pronounced anti-inflammatory effect than local application.

In our experiments, diclofenac, used as a standard anti-inflammatory agent, exhibited a statistically significant reduction in inflammatory edema two to three hours of inflammation induction, depending on the route of application. The lack of diclofenac efficacy during the first phase of inflammation is consistent with the mechanism of action of NSAIDs, including diclofenac [40]. Compared to diclofenac, *N. nuda* extract—applied both locally (at a higher dose of 25 mg/kg) and systemically (at both 50 mg/kg or 200 mg/kg), produced comparable inhibitory effects on inflammatory edema. Locally applied diclofenac reduced paw edema from the 3rd hour after carrageenan injection, whereas *N. nuda* extract at 25 mg/kg showed greater effectiveness at the 4th hour. Intraperitoneal administration of both diclofenac and *N. nuda* extract at either tested dose significantly inhibited carrageenan-induced edema. During the initial phase of inflammation, the effect of N. nuda extracts on paw edema was stronger than that of diclofenac, however, without statistical significance, whereas at the 4th hour diclofenac was more efficient.

The release of histamine and other pro-inflammatory mediators during the first phase of inflammation can increase vascular permeability around the damaged tissue and lead to edema [41]. In our study, locally applied *N. nuda* extract did not affect carrageenan-induced edema during this initial phase (0–1 h), and the higher dose even slightly increased paw swelling. A possible explanation for this outcome may be an enhancement of vascular permeability and subsequent exudation resulting from local application of the plant extract. However, systemic administration of the *N. nuda* extract appeared to suppress the early phase of carrageenan-induced edema. This effect may involve inhibition of the synthesis, release, and/or activity of inflammatory mediators associated with the initiation of the inflammatory response.

The presence of polyphenols reported as phytochemical constituents of several *Nepeta* species [42,43,44] including *N. nuda* [22]) confers notable antioxidant potential, which contributes to their anti-inflammatory effect [45]. Studies examining the anti-inflammatory activity of polyphenols in rats have shown that carrageenan-induced edema is significantly inhibited during the 1st and 2nd hours after induction but insignificantly during the 3rd hour [16].

Some studies have reported that 1, 8-cineole and 4aα, 7α, 7aβ-nepetalactone possess potent anti-inflammatory activity [46,47]. These compounds, considered among the active constituents likely responsible for the anti-inflammatory effects of *Nepeta* species, are thought to act predominantly during the second phase of the inflammation—precisely when the strongest effects of the *N. nuda* extract were observed in our experiments, regardless of the route of administration.

Inflammatory pain is among the various symptoms, induced by the release of inflammatory mediators [48]. Spontaneous motor activity is a reliable indicator for predicting the analgesic efficacy of compounds in inflammatory pain models [28]. To objectively assess the nociceptive behavior (without applying external stimuli) in acute inflammatory pain, we used the carrageenan-induced locomotor activity impairment model in the rat [49]. Carrageenan-induced paw inflammation and pain are expected to impair mobility, which can be quantified as horizontal (ambulatory) or vertical (rearing) locomotion [50]. In our experimental protocol, we evaluated the exploratory behavior of the rats at the peak of inflammation (3 h after carrageenan injections). Since the duration of immobility can be considered as an index of motor impairment, discomfort, and pain, the frequency of horizontal and vertical movements was used to determine the effect of the *N. nuda* extract on the induced inflammatory pain. Regarding the ambulatory activity, only locally administered diclofenac significantly increased the number of horizontal movements, likely reflecting its strong ability to reduce the paw swelling and inflammation at the 3rd hour after carrageenan injection. Intraperitoneally applied, *N. nuda* extract at both tested doses, as well as diclofenac at 25 mg/kg, did not significantly alter horizontal activity of the rats with carrageenan-induced edema.

Quantification of vertical activity is particularly informative, when the lower limbs are injured, since it reflects the rodent’s ability to straighten to its hind legs, a position which maximizes the mechanical pressure of body weight on the hind limbs [51]. Because inflammation was induced in the right hind paw, changes in vertical activity directly reflected the functional status of the injured limb. Locally administered, *N. nuda* extract at 25 mg/kg significantly increased the number of vertical movements suggesting a reduction in pain-related motor discomfort. This positive effect was even stronger than that of the locally administered diclofenac at 0.25 mg/rat. The lower dose of *N. nuda* extract locally applied only slightly, non-significantly increased the vertical locomotion. Systemically administered *N. nuda* extract did not significantly affect vertical activity at either tested dose. Intraperitoneally applied diclofenac increased the rat vertical activity, although with no statistical significance. The efficacy of the topically applied *N. nuda* extract supports the notion that its analgesic effect is at least partly mediated through peripheral mechanisms similar to those of NSAIDs, including prevention of nociceptor sensitization via inhibition of COX metabolites synthesis [52]. Several metabolites present in *N. nuda* such as 1, 8-cineole and 4aα, 7α, 7aβ-nepetalactone have been shown to possess analgesic effects [18,31,53,54,55]. Liapi and coworkers [56] even reported that 1,8-cineole exhibited antinociceptive activity comparable to that of morphine in thermal analgesic stimuli in rats.

Despite the significant anti-inflammatory and analgesic effects observed in this study, the exact central and/or peripheral mechanisms through which *N. nuda* extract acts remain to be elucidated and further detailed investigations are warranted.

## 5. Conclusions

The present study was focused on the in vivo assessment of the potential of *N. nuda* aqueous extract to reduce the inflammation and the related inflammatory pain in order to upgrade the existing data from in vitro experiments. Additionally, the efficiency of systemic and local administration of the extract was evaluated. Our results revealed that the *N. nuda* extract significantly inhibited carrageenan-induced edema following both local and systemic administration in rats. Intraperitoneal administration of *N. nuda* extract induced a stronger anti-inflammatory response compared with the local application.

The higher dose of locally administered *N. nuda* extract showed significant suppression of the inflammation pain, as demonstrated by improved vertical motor activity of the rats. Overall, additional research is needed to elucidate the precise mechanisms and to identify the specific plant metabolites responsible for these effects.

## Figures and Tables

**Table 1:** Extract yield and total quantity of phenolic antioxidants in aqueous extracts from *N. nuda* flowers, leaves and stems.

	Extract Yield %	Phenols mg g DW^−1^	Flavonoids mg g DW^−1^	DPPH mM DW^−1^
Flower	16.6 ^b^	70.24 ^a^	40.26 ^c^	652.51 ^a^
Leaf	19.7 ^a^	63.67 ^b^	55.26 ^a^	391.82 ^c^
Stem	11.9 ^c^	60.06 ^c^	43.16 ^b^	437.41 ^b^

Statistical differences among flower, leaf and stem were determined using one-way ANOVA (Holm–Sidak test), as different letters denote significant variations.

**Table 2:** *p*-values of the comparisons of the paw volumes at each of the time points to the corresponding initial volumes.

Group	*p*-Values			
	Time After Injection, min			
	60	120	180	240
Control (0.9% NaCl i.pl.)	0.1269	0.0504	0.2992	0.6337
Carrageenan (1%, i.pl.)	0.0007	0.0001	0.0001	0.0004
Placebo (0.9% NaCl i.p. + 1% Carrageenan i.pl.)	0.00005	0.0002	0.0002	0.0001

The edema progression in a single animal was analyzed using Paired samples *t*-test as the paw volumes at every of the time points (60, 120, 180, and 240 min after the injections with carrageenan or 0.9% NaCl, respectively) were compared to the corresponding initial volumes. i.pl.—intraplantarly; i.p.—intraperitoneally.

## Data Availability

The original contributions presented in this study are included in the article/Supplementary Materials. Further inquiries can be directed to the corresponding authors.

## Supplementary Materials

The following supporting information can be downloaded at: https://www.mdpi.com/article/10.3390/life15121938/s1, Table S1. Primary and secondary metabolites identified by GC-MS analysis in *N. nuda* flowers [22]; Table S2. Secondary metabolites identified by Orbitrap-MS^n^ analysis in *N. nuda* flowers [20]; Table S3. Metabolic composition in aqueous extracts of *N. nuda* flowering plants. Metabolites identified by NMR analysis are shown [19]; Table S4. Data for the in vitro biological activity of aqueous extract from *N. nuda* flowers.

## Abbreviations

The following abbreviations are used in this manuscript:

NSAIDs	Nonsteroidal anti-inflammatory drugs
COX	Cyclooxygenase
AU	Arbitrary unit
AUC	Area under the curve
