# Supplementary material for: In Vivo Assessment of Anti-Inflammatory Effects of Aqueous Extracts of Nepeta nuda ssp. nuda L. in Experimental Model of Peripheral Inflammation in Male Long Evans Rats
Source: Life (Basel). 2025 Dec 18;15(12):1938. doi: 10.3390/life15121938 (PMC12734412; doi:10.3390/life15121938)
Supplement: Supplementary file 1 [file life-15-01938-s001.zip › life-4012577-supplementary.pdf]

Supplementary Materials

Table S1. Primary and secondary metabolites identified by GC-MS analysis in *N. nuda* flowers [22].

Polar metabolites	*Non-polar metabolites*	
Organic acids	***Fatty acids***	***Essential oils***
Citramalic acid	Docosanoic acid (Behenic acid)	β-Pinene
Citric acid	Eicosanoic acid (Arachidic acid)	Sabinene
Erythronic acid	Heptadecanoic acid (Margaric acid)	Myrcene
Fumaric acid	Hexadecanoic acid, 14-methyl-	D-Limonene
Fumaric acid, 2-methyl- (Mesaconic acid)	Linoleic acid	1,8-Cineole/Eucalyptol
Glycolic acid	Linolenic acid	β-Ocimene
Malic acid	Methyl 2-hydroxytetracosanoate	3-Octanone
Malonic acid	Methyl stearate	Benzene, m-di-tert-butyl-
Methylsuccinic acid	Oleic acid	1-Octen-3-ol
Succinic acid	Palmitic acid	γ-Elemene
Tartaric acid	Pentadecanoic acid	β-Bourbonene
Amino acids	Pentadecanoic acid, 14-methyl-	Camphor
L-Alanine	Stearic acid	α-Gurjunene
γ-Aminobutanoic acid (GABA)	Tetracosanoic acid (Lignoceric acid)	β-Copaene
L-Aspartic acid	***Alkanes***	β-Elemene
Glycine	Dotriacontane	Caryophyllene
L-Glutamic acid	Eicosane	Humulene
Homoserine	Heptadecane	δ-Terpineol
	Hexadecane	α-Terpineol
L-Isoleucine	Octadecane	Germacrene D
L-Leucine	Pentacosane	Bicyclogermacren
Oxoproline	Triacontane	4a-α,7-β,7a-α-Nepetalactone
Proline	***Branched alkanes***	
Serine	3-Methylpentacosane	
Threonine	2-Methyltetracosane	
Alcohols	3-Methyltricosane	
Galactinol	***Sterols***	
Glycerol	α-Amyrin	
Sugar derivatives	β-Sitosterol	
Fructose	Oleanolic acid	
Galactose	Ursolic acid	
Glucose		
Isomaltose		
Mannose		
Myo-Inositol		
Sucrose		
Trehalose, alpha,alpha'-		
Trehalose, beta,beta		
Xylose		
Phenolic derivatives		
Benzoic acid		
Caffeic acid		
Catechollactate/Danshensu		
4-Coumaric acid		
2,5-Dihydroxybenzoic acid (Gentisic acid)		
Homovanillyl alcohol		
Hydroquinone		
Isoferulic acid		
Rosmarinic acid		
Shikimic acid		
Syringic acid		
Tyrosol		
Vanillic acid		
Others		
Quinic acid		

Table S2. Secondary metabolites identified by Orbitrap-MS^n analysis in *N. nuda* flowers [20].

Phenolic acid derivatives	***Flavonoids***	***Iridoid glycosides***
Aesculetin	Apigenin	Epideoxyloganic acid 1
Aesculin	Apigenin 7-*O*-(acetyl)hexuronide	Epideoxyloganic acid 2
Benzoyl tartaric acid	Apigenin 7-*O*-(acetyl-caffeoyl)hexuronide	Epideoxyloganic acid 3
Caffeic acid	Apigenin 7-*O*-(caffeoyl)hexuronide	Epideoxyloganic acid hexoside 1
Caffeic acid hexoside 1	Apigenin 7-*O*-(feruloyl)hexuronide 1	Epideoxyloganic acid hexoside 2
Caffeic acid hexoside 2	Apigenin 7-*O*-(feruloyl)hexuronide 2	Epideoxyloganic acid pentoside
Caffeic acid hexuronide	Apigenin 7-*O*-(sinapoyl)hexuronide	Geniposidic acid
Caffeoyl tartaric acid	Apigenin 7-*O*-hexoside	Loganic acid
Clinopodic acid A	Apigenin 7-*O*-hexuronide	Nepetanudoside
Dihydroxybenzoic acid hexoside 1	Apigenin 7-*O*-hexuronide methyl ester	Nepetanudoside B
Dihydroxybenzoic acid hexoside 2	Apigetrin	
Dihydroxybenzoic acid hexoside 3	Astragalin	
Ethyl caffeate	Cirsimaritin	
Ferulic acid	Galangin	
Feruloyl tartaric acid	Isoquercetin	
Gallic acid hexoside 1	Luteolin	
Gallic acid hexoside 2	Luteolin-7-O-diglucuronide	
Gentisic acid	Luleolin 7-*O*-(feruloyl)hexuronide 1	
Methyl 2-hydroxy-3-(3-hydroxy-4-methoxyphenyl) propanoate	Luleolin 7-*O*-(feruloyl)hexuronide 2	
Methyl rosmarinate	Luleolin 7-*O*-hexuronide 1	
Methyl salvianolate C 1	Luleolin 7-*O*-hexuronide 2	
Methyl salvianolate C 2	Luteolin 7-*O*-(caffeoyl)hexuronide	
Nepetoidin B 1	Luteolin 7-*O*-(acetyl)hexuronide 1	
Nepetoidin B 2	Luteolin 7-*O*-(acetyl)hexuronide 2	
p-Hydroxybenzoic acid	Thymusin	
Protocatechuic acid	Xanthomicrol	
Salvianolic acid C		
Rosmarinic acid		
Syringic acid		
Vanillic acid		

Table S3. Metabolic composition in aqueous extracts of *N. nuda* flowering plants. Metabolites identified by NMR analysis [19].

Organic acids
γ-Aminobutanoic acid (GABA)
Acetic acid
Succinic acid
Formic acid
Fumaric acid
Malic acid
Amino acids
Alanine
Glutamine
Leucine
Threonine
Valine
Sugar derivatives
α-Glucose
β-Glucose
Sucrose
Phenolic derivatives
Gallic acid
Vanillic acid
Protocatechuic acid
Caffeic acid
Ferulic acid
Chlorogenic acid
Rosmarinic acid
Cinnamic acid

Table S4. Data for the in vitro biological activity of aqueous extract from *N. nuda* flowers.

Activity	Assessment			References
**Cytotoxicity** (MDBK cell viability measured at 72 h)	**MNC** (mg ml^{-1})	**CC_{50}** (mg ml^{-1})		[20]
	1.5	6.4		
**Antiviral** (against HHV-1 (strain F))	**Cell protection** (%) when the extract is added in MNC	**EC_{50}** (mg ml^{-1})	**SI**	[20]
The extract added simultaneously with inoculation of cell monolayer	81.37	0.599	10.68	
The extract added 1 h after inoculation of cell monolayer	65.62	1.155	5.54	
**Antioxidant**	**EC_{50}** DPPH Radical scavenging (μg ml^{-1} extract)			[20]
	23.53 ± 0.92			
**Anti-inflammatory**	**% inhibition (at 2 mg ml^{-1})**			[22]
	51.31			

* **MDBK** cells—Madine and Darby bovine kidney cells; **MNC** (maximum nontoxic concentration)—the highest extract concentration that did not cause damage or death to the treated MDBK cells; **CC_{50}** (50% cytotoxicity concentration)—the extract concentration required for the reduction in cell viability by 50%; **EC_{50}** (50% effective concentration)—extract concentration inhibiting viral replication by 50% or for radical scavenging activity, respectively; **SI** (Selective Index)—the ratio of CC_{50} and EC_{50} to determine the extract selectivity to a viral target relative to the cell ($SI = CC_{50}/EC_{50}$); **DPPH**—2,2′-diphenyl-1-picrylhydrazyl.
